# Genetic variants in the *FOXO1* and *ZNF469* genes are associated with keratoconus in Sweden: a case-control study

**DOI:** 10.1186/s12886-024-03299-8

**Published:** 2024-01-24

**Authors:** Wolf Wonneberger, Bertil Sterner, Ulrika MacLean, Margareta Claesson, Lena Havstam Johansson, Ingmar Skoog, Madeleine Zetterberg, Anna Zettergren

**Affiliations:** 1 Region of Västra Götaland, Department of Ophthalmology, Sahlgrenska University Hospital, SE-431 80 Mölndal, Sweden; 2 Department of Clinical Neuroscience, Institute of Neuroscience and Physiology, the Sahlgrenska Academy, University of Gothenburg, Gothenburg, Sweden; 3 Gothenburg Vision Rehabilitation Center, Gothenburg, Sweden; 4 Neuropsychiatric Epidemiology Unit, Department of Psychiatry and Neurochemistry, Institute of Neuroscience and Physiology, Sahlgrenska Academy, Centre for Ageing and Health (AgeCap), University of Gothenburg, Mölndal, Sweden

**Keywords:** Sporadic keratoconus, *FOXO1* gene, *ZNF469* gene, Single-nucleotide polymorphism

## Abstract

**Background:**

Keratoconus (KC) is characterized by pathological thinning and bulging of the cornea that may lead to visual impairment. The etiology of sporadic KC remains enigmatic despite intensive research in recent decades. The purpose of this study was to examine the relationship between previously highlighted genetic variants associated with KC and sporadic KC in a Swedish cohort.

**Methods:**

A total of 176 patients (age 16–70 years) with sporadic KC diagnosed by Scheimpflug-topography (Pentacam) were included. The control group (*n* = 418; age 70 years) was a subsample originating from the Gothenburg H70 Birth Cohort Studies of ageing. Extraction of DNA from blood samples was performed according to standard procedures, and genotyping was performed using competitive allele specific PCR (KASP) technology. A total of 11 single nucleotide polymorphisms (SNPs) were selected for analysis.

**Results:**

Statistically significant associations (*p* = 0.005) were found between the SNPs rs2721051 and rs9938149 and sporadic KC. These results replicate earlier research that found associations between genetic variants in the *FOXO1* and *BANP-ZNF469* genes and sporadic KC in other populations.

**Conclusion:**

Genetic variations in the *FOXO1* and *BANP-ZNF469* genes may be involved in the pathogenesis of sporadic KC.

## Background

Keratoconus (KC) is characterized by bilateral but asymmetric, pathological thinning and bulging of the cornea that may lead to visual impairment [1]. With a prevalence of approximately 1 in 2000 in the general population, KC is also a relatively common eye disease [2]. Spectacles, rigid gas-permeable contact lenses and insertion of corneal ring segments are treatment modalities commonly applied to improve visual function. Corneal collagen crosslinking (CXL) aims to halt the progression of KCs. In some cases, corneal transplantation is deemed necessary, thus replacing the thinned part of the cornea with an allogenic transplant. Eye rubbing, improper fit of contact lenses and chronic ocular irritation, have all been found to increase the risk for progression of KC [3]. In most cases, however, the etiology of KC remains enigmatic. The etiology is likely multifactorial, and several observations make a genetic component plausible. Autosomal dominant inheritance in families, higher incidence in families with one affected family member and high concordance among identical twins suggest a genetic cause of the disease [4, 5]. The results of the first genome-wide association study (GWAS) of KC by Li et al. in 2012 suggested an association of the single-nucleotide polymorphism (SNP) rs4954218 (located near the *RAB3GAP1* gene) with the development of sporadic KC [6]. The findings of another GWAS by Burdon et al. indicated possible KC susceptibility through variations in the hepatocyte growth factor (*HGF*) gene [7]. In 2013, a comprehensive GWAS in a Caucasian population performed by Lu et al. identified 16 new loci that were associated with central corneal thickness (CCT), and two of these CCT-associated loci (*FOXO1* and *FNDC3B)* were related to an increased risk of developing KC [8]. *FOXO1* encodes the forkhead box 01 protein which is involved in the regulation of different cellular processes. These include the response of cells to oxidative stress which may contribute to corneal thinning. *FOXO1* may also influence cell proliferation and differentiation which could affect corneal tissue. *FNDC3B* codes for a fibronectin type III protein and variations in this gene may hypothetically also affect cornea tissue leading to thinning and deformation.

The aim of this study was to examine the relationship between previously highlighted genetic variants associated with KC and central corneal thickness (CCT) and sporadic KC in a Swedish cohort.

## Methods

### Patients with sporadic KC

Patients with sporadic KC in this study were examined during the recruitment process of a randomized controlled clinical trial aiming to study the efficacy of CXL in progressive KC. The trial was approved by the regional ethical review board in Gothenburg, Sweden (approval number 949 − 11), and the study followed the terms of the Declaration of Helsinki. Informed written consent was obtained from each patient. Blood samples were drawn from each participant and stored for future analyses according to the Swedish Biobank Law. All study participants gave written consent regarding biobanking.

Inclusion criteria were age of 17 to 35 years and tomographic diagnosis of KC as defined by the Pentacam Scheimpflug camera topometric module (KC grade 1 or higher), the ability to stop contact lens wear (rigid and soft) at least two weeks prior to exam, and that three consecutive, topographic exams of each eye with approved quality rating at first and second visits could be obtained.

### Diagnosis of KC in patients with sporadic KC

Both eyes of the patients with sporadic KC were examined with three consecutive tomography images. The measurements were performed by either of two experienced examiners (BS, UM). The tomography instrument was calibrated according to the manufacturers’ recommendations. All measurements were performed under scotopic light conditions. The patient had both eyes open during measurements. The patient fixated with the eye to be measured at the red fixation light of the Pentacam system and was instructed to blink before every measurement. The automated release mode with 25 pictures was used. The Pentacam KC staging system was used to diagnose KC in combination with individual assessment of the tomography results by a corneal surgeon (WW) with regard to the plausibility of the diagnosis. The tomographical diagnosis of KC was based on keratometry values, minimal corneal thickness, typical distribution of corneal thickness in relation to keratometry values and presence of pathological elevations of the anterior and / or poster corneal surface. Generally, keratometry values above 48 diopters and posterior corneal elevations of more than 35 μm were considered as pathological. The diagnosis of KC, however, also weighed in the overall picture as described above. All patients underwent a complete slit-lamp exam.

### Controls

The Gothenburg H70 Birth Cohort Studies examine cohorts of 70-year-olds with the aim of learning more about aging. The participants in the control group in our study were chosen from a cohort of individuals born in 1944 who participated in the H70 study during 2014–2016 [9]. A subsample of 560 patients underwent extended ophthalmic examinations [10]. The H70 study was approved by the regional ethical review board, and informed, written consent, including biobankning, was obtained from each patient.

### Absence of corneal ectasia in the controls

All participants in the control group underwent comprehensive ophthalmic examination, including objective refraction with an auto kerato-refractometer. Individuals with central corneal astigmatism below 1.5 diopters (D) were chosen as the control group under the assumption that the likelihood for KC was sufficiently low, especially since they were all 70 years of age and the disease rarely manifests itself or progresses after the age of 30.

### SNP selection and genotyping

The selection of SNPs (i.e. *FOXO1*: rs2721051, *COL5A1*: rs7044529, *RXRA-COL5A1*: rs7044529, *FINDC3B*: rs4894535, *BANP-ZNF469*: rs9938149, *RAB3GAP1*: rs4954218, and *HGF*: rs2286194, rs17501108, and rs3735520) were based on findings in previous GWASs on KC and CCT [6–8]. Furthermore, SNPs in the *LOX* gene (rs10519694 and rs2956540) that have been associated with KC in several previous case-control studies were selected [11, 12]. Extraction of DNA from blood samples was performed according to standard procedures. Genotyping was performed at LGC Genomics (Hoddesdon, Herts, UK) using competitive allele specific PCR (KASP) technology. The success rate was over 95% for all genotyped SNPs, and they were in Hardy-Weinberg equilibrium. One of the selected SNPs (rs1324183 in *MPDZ-NF1B*) was not genotyped due to unsuccessful design of the KASP assay.

### Statistical analysis

IBM SPSS Statistics software (version 28.0, IBM, Armonk, NY, USA) was used for statistical analysis. As descriptive statistics, means and standard deviations (SD) were calculated as well as proportions for categorical data. Fisher’s exact test was used for statistical analysis of the latter. Student’s t-test was used for statistical analysis of continuous data. A *p*-value < 0.05 was considered statistically significant.

Associations between sporadic KC and genetic variants were analyzed using logistic regression models. Two different models were tested: an additive genetic model (i.e. Rare genotypes are coded as 2, heterozygotes are coded as 1, and common genotypes are coded as 0) and a dominant genetic model (i.e. Rare genotypes and heterozygotes are coded as 1, and common genotypes are coded as 0. Only individuals of Caucasian origin (163 patients and 406 controls) were included in the analyses. The analyses were also repeated after excluding individuals born outside of Europe (including 153 patients and 399 controls in the analyses).

Post hoc power was calcualted using the G*Power software (version 3.1.9.7), including the effect sizes found in the genetic association analyses (dominant model) and a *p*-value level of 0.05.

## Results

The sample characteristics of the KC patients and the control individuals are shown in Table 1. Significant differences were seen for sex and age between the two groups (*p* < 0.001). Notably, in the KC group, only 11% were women.

**Table 1:** Baseline characteristics of the studied individuals

Cohort	KC	H70	*p*
Characteristics	N	N	
Number of patients,	176	418	
Females (%)	20 (11)	226 (54)	**< 0.001**
Males (%)	156 (89)	192 (46)	
Age, years (mean ± SD)	27 ± 7	70	**< 0.001**
Age, years, range	16–70	N.A.	
Ethnicity	Caucasian	Caucasian	

N, numbers except for age

The results for the genetic analysis are summarized in Table 2. Statistically significant associations were found between the SNPs rs2721051 in *FOXO1* and rs9938149 in *BANP-ZNF469* and sporadic KC. For rs2721051, the A allele was associated with an increased risk of disease, and for rs9938149, the C allele was associated with a decreased risk of disease (i.e. the C allele contributed with a protective effect). These associations were seen both in the additive and dominant models. None of the other investigated SNPs were associated with KC in our sample. When repeating the analyses after excluding non-European individuals, the associations with the two SNPs in *FOXO1* and *BANP-ZNF469* remained (results not shown). The allele frequencies of these two SNPs in the control population were similar to those reported for other European samples in the genetic data base dbSNP [13].

**Table 2:** Summary for the genetic analysis

Gene: SNP	Genotype	Cases	Controls	OR (95% CI)^add^	p^add^	OR (95% CI)^dom^	p^dom^
	n (%)	n (%)					
*FOXO1*:rs2721051	AA	4 (2.5)	11 (2.7)	1.90 (1.27–2.40)	0.002*	2.32 (1.44–3.74)	**0.005**
	AG	44 (27.2)	56 (13.9)				
	GG	114 (70.4)	337 (83.4)				
*BANP-ZNF469*:rs9938149	CC	15 (9.3)	42 (10.4)	0.66 (0.48–0.89)	**0.008**	0.53 (0.35–0.79)	0.002*
	CA	56 (34.8)	201 (49.8)				
	AA	90 (55.9)	161 (39.9)				
*COL5A1*:rs7044529	TT	2 (1.2)	7 (1.7)	1.19 (0.81–1.75)	0.4	1.28 (0.84–1.95)	0.2
	TC	56 (34.8)	111 (27.4)				
	CC	103 (64.0)	287 (70.9)				
*RXRA-COL5A1*:rs1536482	AA	16 (9.9)	42 (10.4)	1.14 (0.84–1.54)	0.4	1.16 (0.78–1.73)	0.5
	AG	70 (43.2)	154 (38.0)				
	GG	76 (46.9)	209 (51.6)				
*FINDC3B*:rs4894535	TT	6 (3.7)	15 (3.7)	0.75 (0.52–1.08)	0.1	0.68 (0.45–1.04)	0.07
	TC	45 (27.6)	130 (32.0)				
	CC	112 (68.7)	261 (64.3)				
*RAB3GAP1*:rs4954218	GG	11 (6.8)	38 (9.4)	0.88 (0.64–1.19)	0.4	0.88 (0.59–1.31) 0.5	0.5
	GT	64 (39.6)	169 (41.9)				
	TT	87 (53.7)	196 (48.6)				
*HGF*:rs2286194	AA	7 (4.3)	12 (3.0)	1.13 (0.64–1.19)	0.5	1.14 (0.75–1.74) 0.5	0.5
	TA	47 (29.2)	110 (27.1)				
	TT	107 (66.5)	284 (70.0)				
*HGF*:rs3735520	AA	31 (19.4)	83 (20.4)	0.96 (0.73–1.28)	0.8	0.93 (0.61–1.42)	0.7
	GA	77 (48.1)	197 (48.5)				
	GG	52 (32.5)	126 (31.0)				
*HGF*:rs17501108	TT	1 (0.6)	9 (2.2)	0.69 (0.45–1.06)	0.09	0.71 (0.44–1.13) 0.2	0.2
	TG	33 (20.5)	94 (23.3)				
	GG	127 (78.9)	301 (74.5)				
*LOX*:rs2956540	GG	33 (20.2)	90 (22.4)	0.82 (0.62–1.07)	0.1	0.78 (0.51–1.20)	0.3
	GC	75 (46.0)	197 (49.0)				
	CC	55 (33.7)	115 (28.6)				
*LOX*:rs10519694	TT	10 (6.2)	35 (8.6)	0.80 (0.58–1.09)	0.2	0.80 (0.54–1.19)	0.3
	TC	64 (39.5)	166 (40.9)				
	CC	88 (54.3)	205 (50.5)				

SNP, single nucleotide polymorphism; n, numbers; OR, Odds Ratio; add, additive genetic model (i.e. rare genotypes are coded as 2, heterozygotes coded as 1, and common genotypes coded as 0); dom, dominant genetic model (i. e. rare genotypes and heterozygotes are coded as 1, and common genotypes coded as 0). Significant *p*-values are shown in bold. A *p*-value < 0.05 was considered significant. Results are based on logistic regression models adjusted for sex. *p-values surviving Bonferroni correction for multiple testing based on 11 independent tests

Post hoc power to detect a significant finding showed a power of 81–100% for five SNPs (*FOXO1*: rs2721051, *BANP-ZNF469*: rs9938149, *COL5A1*: rs7044529, *HGF*: rs17501108, and *LOX*: rs2956540), a power of 50 to 71% for five SNPs (*RXRA-COL5A1*: rs7044529, *FINDC3B*: rs4894535, *HGF*: rs2286194, and *LOX*: rs10519694) and a power of 13% for one SNP (*HGF*: rs3735520).

## Discussion

For the first time in a Swedish cohort, our study showed associations of two SNPs with sporadic KC. The SNPs were located in the genes *FOXO1* and *BANP-ZNF469*.

As outlined earlier, the selection of SNPs was based on findings in previous GWASs on KC and CCT [6–8]. SNPs in the *LOX* gene (rs10519694 and rs2956540) that have been associated with KC in several previous case-control samples were also selected [11, 12].

*FOXO1* is a member of the forkhead box (Fox) transcription factor family, and Fox proteins regulate cellular oxidative stress [14]. Increased oxidative stress may damage the trabecular meshwork and lead to elevated intraocular pressure and corneal thinning [15]. In both primary open-angle glaucoma (POAG) and KC, CCT is known to be reduced [16, 17]. In 2010, Lu et al. identified a SNP near *FOXO1* (rs2721051), which accounted for approximately 1.2% of the variation in normal CCT [18]. Another study by Lu et al. found a strong association between KC risk and *FOXO1* (OR = 1.62, 95% CI 1.4–1.88, *P* = 2.7 × 10^− 10^), which is comparable to the findings in our study [8].

Rare variants in the *ZNF469* gene have been reported to be involved in the development of brittle cornea syndrome (BCS), which leads to thinning of the cornea and progressive visual impairment [19]. Common variants near *ZNF469* were associated with CCT according to Lu et al. [18]. Furthermore, based on the finding of a KC locus close to the location of *ZNF469* in the Finnish family, Tyynismaa et al. argued that mutations in the *ZNF469* gene might be found in patients with KC [20]. The role of *ZNF469* in the development of KC is, however, not clear. Regarding common genetic variants, our study confirmed the findings by Lu et al., showing that the CCT-reducing allele near *ZNF469* (rs9938149), actually, is associated with a decreased KC risk [8].

Very recently, Hardcastle et al. reported the results of the first large-scale genome-wide association study of KC [21]. This multicenter study included 4669 cases and 116,547 controls and revealed genome-wide significant associations for 36 genomic loci, including those found to be associated with KC in our study (i.e. *FOXO1* and *ZNF 469*). The study clearly supports a genetic component of KC. It was estimated that 12.5% of the heritability of KC in European individuals was explained by the common variants associated with KC in this study.

Among the limitations of our study is that exclusion of KC in the control group was not based on topographic examinations. The effect of this limitation should, however, be small. The prevalence of sporadic KC is rather low, and individuals with central corneal astigmatism above 1.5 diopters were excluded. The high age of the control subjects, 70 years, also precludes later development of the disease. Another limitation is that due to a relatively small sample size only five SNPs reached a power of over 80%, when post hoc power to detect associations were calculated. It is, however, important to note that for some SNPs very small effect sizes were used in the power calculations, since we included the ORs found for each specific SNP. For some of the SNPs, such as those in the *LOX* gene, the effect sizes (i.e. ORs) in our study are of comparable size to previous studies [22], but the *p*-values do not reach the level of significance. However, many of the nonsignificant results in our study show an effect size below the findings in previous studies, indicating that these genetic variants are of low importance in relation to KC in our sample [22].

Finally, it is well known that KC is more common in males [23]. This is also reflected in the significant difference between the number of males in the study group and the control group in this study. This might affect the significance of the results. It is, therefore, important to point out the that the results in this study are based on logistic regression models adjusted for sex.

## Conclusion

This study demonstrates associations between two SNPs in *FOXO1* and *BANP-ZNF469* and KC in a Swedish sample. The results corroborate previously reported associations between these genes and sporadic KC.

## Data Availability

The datasets used and/or analysed during the current study are available from the corresponding author on reasonable request. Confidential data will, however, not be shared.
